# A connectivity-based test-retest dataset of multi-modal magnetic resonance imaging in young healthy adults

**DOI:** 10.1038/sdata.2015.56

**Published:** 2015-10-27

**Authors:** Qixiang Lin, Zhengjia Dai, Mingrui Xia, Zaizhu Han, Ruiwang Huang, Gaolang Gong, Chao Liu, Yanchao Bi, Yong He

**Affiliations:** 1 State Key Laboratory of Cognitive Neuroscience and Learning & IDG/McGovern Institute for Brain Research, Beijing Normal University, Beijing 100875, China

**Keywords:** Bioinformatics, Magnetic resonance imaging, Brain imaging, Functional magnetic resonance imaging

## Abstract

Recently, magnetic resonance imaging (MRI) has been widely used to investigate the structures and functions of the human brain in health and disease *in vivo*. However, there are growing concerns about the test-retest reliability of structural and functional measurements derived from MRI data. Here, we present a test-retest dataset of multi-modal MRI including structural MRI (S-MRI), diffusion MRI (D-MRI) and resting-state functional MRI (R-fMRI). Fifty-seven healthy young adults (age range: 19–30 years) were recruited and completed two multi-modal MRI scan sessions at an interval of approximately 6 weeks. Each scan session included R-fMRI, S-MRI and D-MRI data. Additionally, there were two separated R-fMRI scans at the beginning and at the end of the first session (approximately 20 min apart). This multi-modal MRI dataset not only provides excellent opportunities to investigate the short- and long-term test-retest reliability of the brain’s structural and functional measurements at the regional, connectional and network levels, but also allows probing the test-retest reliability of structural-functional couplings in the human brain.

## Background & Summary

The human brain is considered to be the most complex system in the nature which is structurally and functionally organized, thus enabling the segregation and integration of efficient information processing. Advances in non-invasive multi-modal magnetic resonance imaging (MRI) techniques have allowed researchers to map and analyze the structures and functions of the human brain at macro-scale *in vivo*^1–6^. Specifically, these imaging measurements have been found to be able to capture brain structural and functional changes in development^7,8^, aging^8,9^ and in neurological and psychiatric disorders such as Alzheimer’s disease^10–14^, mild cognitive impairment^12,13^, schizophrenia^15^ and depression^16,17^. More importantly, these imaging-based measurements have great potential to serve as imaging biomarkers of normal development, aging and the clinical diagnosis and therapeutic assessment of many neurological and psychiatric disorders^12–20^.

A crucial prerequisite for the successful application of these imaging measures is high test-retest (TRT) reliability. In fact, there are growing concerns about the TRT reliability of these MRI measurements and a number of studies have been published to investigate the TRT reliability in either functional or structural measurements^21–27^. Recently, an influential open-access TRT dataset of functional connectomics has been released by the Consortium for Reliability and Reproducibility (CoRR)^28^, which has aggregated TRT datasets from over 18 institutions worldwide. Most of these datasets, however, include only one or two modalities of MRI.

Here, we present a TRT dataset of multi-modal MRI that includes structural MRI (S-MRI), diffusion MRI (D-MRI) and resting-state functional MRI (R-fMRI) with short- and long-term TRT data and has been shared as part of the CoRR consortium^28^. This TRT dataset is a subset of the Connectivity-based Brain Imaging Research Database (C-BIRD) at Beijing Normal University. Notably, the C-BIRD contains 147 young healthy subjects with multi-modal MRI data and a series of behavior test data involving language tests, classical cognitive tests and several other emotion-related questionnaires, which are not released here. This TRT dataset provides excellent opportunities to investigate the short- and long-term TRT reliability of the brain’s structural and functional measurements. For example, using the S-MRI data, researchers can study the TRT reliability of regional morphological measurements (e.g., grey matter volume, cortical thickness and surface area). Using the D-MRI data, researchers can study the TRT reliability of local diffusion measures [e.g., fractional anisotropy (FA)^29^, mean diffusivity (MD)^29^ and local diffusion homogeneity (LDH)^30^] and graph-based network measurements (e.g., cluster coefficient, shortest path length, global and local efficiency and network hubs)^31–33^. Using the R-fMRI data, researchers can investigate the short- and long-term TRT reliability of regional [e.g., amplitude of low-frequency fluctuation (ALFF)^34^, fractional ALFF (fALFF)^35^, and regional homogeneity (ReHo)^36^], connectivity (e.g., seed-based functional connectivity^37^ and independent component analysis^38^) and graph-based network metrics (e.g., cluster coefficient, shortest path length, global and local efficiency and network hubs)^31–33^. This multi-modal MRI dataset also allows probing the test-retest reliability of structural-functional couplings in the human brain at the regional, connectivity and network levels. Recently, this dataset has been used to investigate the construction of structural and functional connectomes^39,40^ and the TRT reliabilities of graph-based functional network metrics^26,27^.

In this data descriptor, we present the details of our TRT dataset and a computed series of quality metrics for the raw MRI data. We have also assessed the TRT reliability of a group of basic imaging measures derived from the multi-modal MRI data, with a particular focus on head motion measures in R-fMRI.

## Methods

### Participants

Fifty-seven healthy young adult volunteers (male/female: 30/27, age: 23.05±2.29; age range: 19–30) were recruited from the campus of Beijing Normal University by advertising on the Bulletin Board System. All participants gave their written informed consent for participation in this study and agreed to freely share of the data on the internet in anonymous form. This study was approved by the Institutional Review Board (IRB) of the State Key Laboratory of Cognitive Neuroscience and Learning at Beijing Normal University.

The inclusion criteria of participants were as follows: (1) Age between 19 and 30 years; (2) Right handed; (3) Native Chinese speakers; (4) Healthy; (5) Undergraduate or postgraduate students, excluding college freshmen; and (6) Not majoring in psychology or with a psychological academic background.

The exclusion criteria of participants were as follows: (1) Metal or implanted devices such as artificial teeth or cardiac pacemaker within the body; (2) Claustrophobia; (3) History of head trauma; or (4) Participants or their first- and second-degree relatives with a history of neurological and psychiatric disorders.

Each participant completed two separated scan sessions at an interval of approximately 6 weeks (40.94±4.51 days). The first scan session (S1) included one each of S-MRI, T2-weighted structural imaging and D-MRI and two R-fMRI scans. The two R-fMRI scans were performed at the beginning (S1-1) and the end (S1-2) of the session (approximately 20 min apart). The second scan session (S2) included S-MRI D-MRI and R-fMRI.

To ensure the participants were in good conditions during the MRI scan, we carefully provided them with detailed instructions and requirements and communicated with them in person before the experiments (Table 1).

### MRI acquisition

All MRI data were obtained using a SIEMENS Trio Tim 3.0 T scanner (Siemens Healthcare, Erlangen Germany) with a 12-channel phased-array head coil in the Imaging Center for Brain Research, Beijing Normal University. The imaging protocols used for MRI acquisition are parameter-optimized protocols and the details of the acquisition parameters for each MRI sequences are summarized below.

### S-MRI data

Structural MRI data were acquired using a T1-weighted, sagittal 3D magnetization prepared rapid gradient echo (MP-RAGE) sequence. The sequence parameters were repetition time (TR)=2,530 ms, echo time (TE)=3.39 ms, inversion time (TI)=1,100 ms, flip angle=7°, field of view (FOV)=256×256 mm, in-plane resolution=256×256, slice thickness=1.33 mm, voxel size=1 mm×1 mm×1.33 mm and 144 interleaved sagittal slices covering the whole brain; acquisition time: 8:07 (m:ss).

### D-MRI data

Diffusion weighted imaging data were acquired using a single-shot twice-refocused spin-echo diffusion echo-planar imaging (EPI) sequence with implementation of the parallel imaging scheme GRAPPA (GeneRalized Autocalibrating Partially Parallel Acquisitions) and an acceleration factor of 2. The sequence parameters were TR/TE=8,000 ms/89 ms, 30 non-linear diffusion directions with b=1,000 s mm^−2^ and an additional volume with b=0 s mm^−2^, in-plane matrix size=128×128, field of view (FOV)=282 mm×282 mm, 2.2 mm slice thickness, isotropic voxel size (2.2 mm^3^), bandwidth (BW)=1562 Hz per pixel, and 62 interleaved transverse slices without gap covering the whole brain and two averages; acquisition time: 4:34 (m:ss) ×2.

### R-fMRI data

The R-fMRI data were obtained using a T2*-weighted echo-planar imaging (EPI) sequence with the following parameters: TR/TE=2,000 ms/30 ms, flip angle=90°, 33 interleaved axial slices, slice thickness/gap=3.5/0.7 mm, in-plane resolution=64×64, FOV=200 mm×200 mm, voxel size=3.125 mm×3.125 mm×4.2 mm; 200 volumes; acquisition time: 6:46 (m:ss).

## Data Records

This dataset has been shared as part of the Consortium for Reliability and Reproducibility (CoRR)^28^. According to prior FCP/INDI policies, the facial information of each participant has been removed from the S-MRI data (FullAnonymize.sh V1.0b; http://www.nitrc.org/frs/shownotes.php?release_id=1902), and the Neuroimaging Informatics Technology Initiative (NIFTI) headers have been replaced before open sharing to minimize the risk of re-identification.

All data are freely available from the INDI/CORR consortium (Data Citation 1). There is a brief description and a detailed scan parameter document for this dataset at this URL. MRI data of all participants are stored in three compressed tar (.tar.gz) files and a comma separated value (.csv) file that contains basic phenotypic information such as gender and age at first scan 1. Each compressed tar file consists of the MRI data of approximately 20 participants. The MRI data were stored in NIFTI format (.nii) in each participant’s data folder. There is a csv file named CorrDataLegend.csv that includes the detailed descriptions of each column field of the phenotypic data. (http://fcon_1000.projects.nitrc.org/indi/CoRR/Data/CorrDataLegend.csv). The full phenotypic information for each participant and the results of quality control measures are also available from our group website (http://www.yonghelab.org/downloads/data).

## Technical Validation

MRI images of each participant were visually inspected immediately after the acquisition of each modality to check for severe motion artefacts or any other apparent artefacts. The S-MRI and T2-weighted structural images of each participant were inspected by two experienced radiologists from the Beijing Xuanwu Hospital of Capital Medical University and no abnormalities were found in any participant’s structural images.

**Figure 1:**
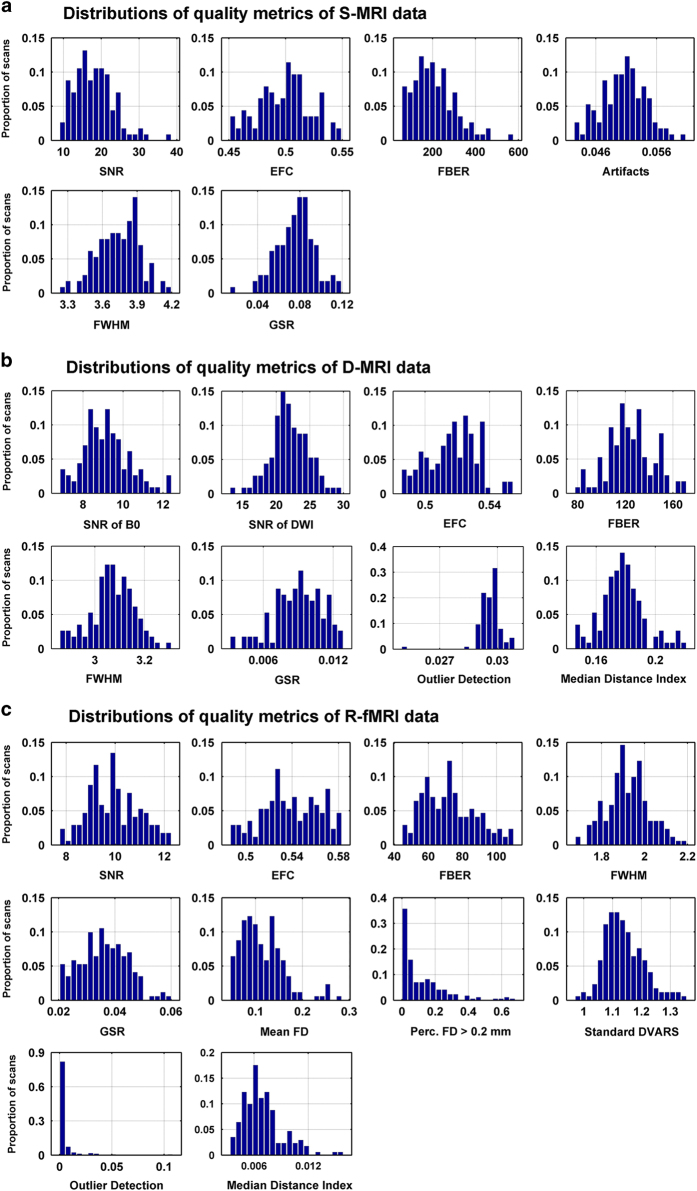
Distributions of quality metrics of MRI data. Y axis indicates the proportion of scans. (**a**) Distributions of quality control metrics of S-MRI data. (**b**) Distributions of quality metrics of D-MRI data. (**c**) Distributions of quality metrics of R-fMRI.

To assess the quality of MRI data, we have calculated a series of quality metrics (Fig. 1) that have been used in previous imaging literatures for each modality of MRI images. All of these metrics were computed using the Preprocessed Connectomes Project Quality Assessment Protocol (http://preprocessed-connectomes-project.github.io/quality-assessment-protocol). Most of these metrics were the same as the ones used in the CoRR paper^28^. The details for the calculation of these metrics are described below.

### Spatial metrics

#### Signal-to-noise ratio (SNR)

The mean values within the brain divided by the standard deviation of the air values^41^. Higher SNR values represent better signal.

#### Foreground to background energy ratio (FBER)

Mean energy of image values (i.e., mean of squares) within the head relative to outside the head. Higher FBER values mean clearer signals.

#### Entropy focus criteria (EFC)

Shannon’s entropy is used to summarize the principal directions distribution; higher energy indicates that the distribution is more uniform (i.e., less noisy)^42^.

#### Smoothness of voxels

The full-width half maximum (FWHM) of the spatial distribution of intensity values of the MRI image^22^.

#### Ghost to signal ratio (GSR)

A measure of the mean signal in the ‘ghost’ image (signal present outside the brain due to acquisition in the phase encoding direction) relative to the mean signal within the brain^43^. Lower values indicate fewer ghost artefacts.

#### Artifact detection (only for S-MRI data)

The proportion of voxels with intensity corrupted by artifacts normalized by the number of voxels in the background^44^. Lower values indicate better image quality.

### Temporal metrics

#### Mean framewise displacement (FD) and percent of volumes with FD greater than 0.2 mm (only for R-fMRI data)

Framewise displacement is a measure of subject head motion that compares the motion between adjacent volumes^45^. This value is calculated by summing the absolute value of displacement changes in the x, y and z directions and rotational changes about those three axes. The rotational changes are given distance values based on the changes across the surface of a 50-mm-radius sphere. Lower values mean less motion. FD >0.2 mm is considered as a threshold for scrubbing^46^.

#### Standardized DVARS (only for R-fMRI data)

The spatial standard deviation of the temporal derivative of the data (D referring to temporal derivative of time series, VARS referring to root-mean-square variance over voxels)^45^, normalized by the temporal standard deviation and temporal autocorrelation (http://blogs.warwick.ac.uk/nichols/entry/standardizing_dvars). Lower values are better.

#### Outlier detection (only for R-fMRI and D-MRI data)

The mean fraction of outliers found in each volume using the **3dTout** command in the software package for Analysis of Functional NeuroImages (AFNI: http://afni.nimh.nih.gov/afni)^47^. Lower number of outliers means better quality.

#### Median distance index (MDI) (only for R-fMRI and D-MRI data)

The mean distance (1—spearman’s rho) between each time-point's volume and the median volume using AFNI’s **3dTqual** command. Smaller values mean more homogeneous time series.

### Regional and connectivity metrics

In addition to the quality control metrics above, several regional and connectivity metrics of S-MRI, D-MRI and R-fMRI data were also calculated for each subject. These metrics include:

#### Gray matter volume (GMV) of S-MRI data

Gray matter volume of each voxel within the gray matter mask of the brain^48^. GMV is calculated by using SPM8 software (http://www.fil.ion.ucl.ac.uk/spm/software/spm8/)^,49^. Briefly, T1 images were segmented into gray matter (GM), white matter (WM) and cerebrospinal fluid (CSF) using the new segment option in SPM8. Then, GM images were normalized into the MNI152 space by using the DARTEL algorithm^50^. Finally, modulated GM images were smoothed using an 8-mm FWHM Gaussian kernel.

#### Fractional anisotropy (FA), mean diffusivity (MD), axial diffusivity (AD) and radial diffusivity (RD) of D-MRI data

FA is a measure of the degree of directionality of diffusion tensor within a voxel^29^. MD measures the mean diffusivity of the three directions. AD is the diffusivity of the axial direction and RD is the mean diffusivity of radial directions. All diffusion metrics were calculated using the FDT toolbox of FSL^51^. We also computed the mean FA, MD, AD and RD of 48 WM tracts in the JHU-ICBM-DTI-81 atlas^52^.

#### Functional connectivity strength (FCS) of R-fMRI data

We calculated the average functional connectivity between a given voxel and all other voxels within the grey matter mask in the brain. The details of the calculation procedure of FCS were described in our previous study^25,26,53^. All preprocessing procedures of R-fMRI data were carried out using the SPM8 and the Data Processing Assistant for Resting-State fMRI (DPARSF)^54^. Briefly, the first 10 volumes were deleted before the slice timing correction and head motion correction. Four subjects were excluded from the analysis because three had excess head motion (translation >2 mm or rotation >2°) in at least one scan session and one subject had missing slices in the R-fMRI data, which has been widely used in previous R-fMRI studies. The individual T1-weighted images were coregistered to the mean functional image after motion correction and were then segmented into GM, WM and CSF. The resulting GM and WM images were further nonlinearly registered into MNI space, and the transfer parameters were estimated. These parameters were then used to normalize all the functional images into MNI152 space and the normalized images were further smoothed using a 4-mm FWHM Gaussian kernel. Time series of each voxels was filtered with a band-pass filter (0.01–0.1 Hz). Then, the nuisance signals (6 head motion parameters, global signal, CSF, and WM signals) were regressed out from each voxel’s time course. The residuals were used for the following resting-state functional connectivity analysis. Then, we computed the connectivity by estimating Pearson’s correlations between the time series of any pairs of brain voxels, resulting in an individual whole-brain functional connectivity matrix. This procedure was constrained within the GM mask generated by thresholding (cutoff=0.2) the group mean GM probability map. Individual FCS maps were obtained for each subject. Notably, given the ambiguous physiological meaning of negative connectivity^24,55,56^, we restricted the FCS analysis within positive connectivity.

### Test-retest reliability

To measure the TRT reliability of our dataset, we applied the statistical measure of the intra-class correlation coefficient (ICC)^57^. The ICC is defined as follows:

$$\mathrm{ICC}=\frac{BMS-WMS}{BMS+(k-1)WMS}$$

where *BMS* is the between-subject mean square, *WMS* is the within subject mean square and *k* represents the number of repeated observations per subject. For the voxel-wise metrics, we calculated the ICC value for each voxel and then obtained the ICC map for each metric.

For the S-MRI data, we obtained the ICC maps for GMV within the GM mask. For the D-MRI data, we first obtained the ICC maps for FA, MD, AD and RD, and then calculated the ICC values for FA, MD, AD and RD of each WM tract in JHU-ICBM-DTI-81 atlas. For the R-fMRI data, we obtained the ICC maps for FCS within the GM mask across the three R-fMRI scan sessions.

Recent studies have demonstrated that head motion may reflect individual neurobiological traits rather than artifacts in the R-fMRI analysis^58–60^. In this study, we further assessed the TRT reliability of several head motion measurements, including the mean displacement, mean rotation, mean FD and standard DVARS of R-fMRI data.

### Results of quality metrics of MRI data

Figure 1 shows the distributions of the quality metrics of S-MRI, D-MRI and R-fMRI data across all participants and all sessions.

### Results of test-retest reliability of regional and connectivity metrics

**Figure 2:**
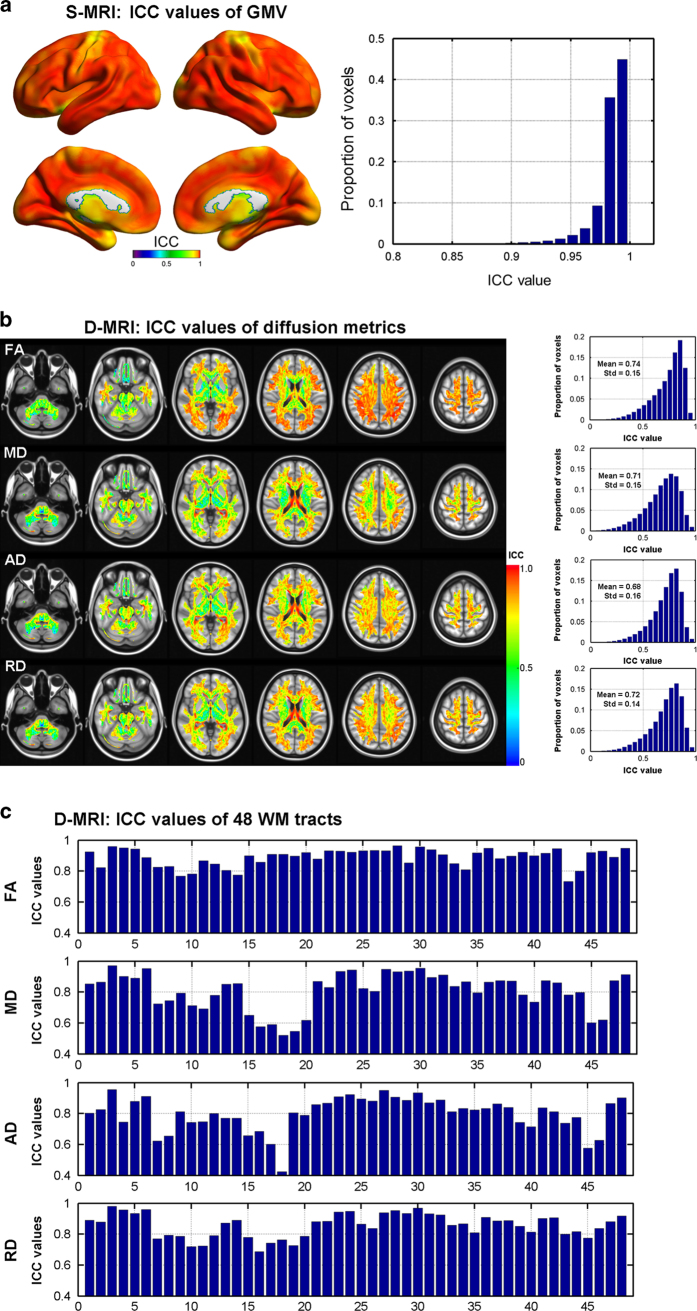
ICC values of GMV and diffusion metrics. (**a**) ICC values of GMV derived from S-MRI data. (**b**) ICC values of four diffusion metrics derived from D-MRI data. (**c**) ICC values of diffusion metrics in the 48 WM tracts of JHU ICBM DTI-81 atlas. The brain figures were visualized using the BrainNet Viewer (http://www.nitrc.org/projects/bnv/)^63^.

Figure 2a illustrates the ICC maps for GMV derived from S-MRI data. The ICC maps of GMV show very high TRT reliability (ICC=0.98±0.03). Figure 2b exhibits the ICC maps for FA, MD, AD and RD derived from D-MRI data. All four diffusion metrics show high TRT reliability within the WM mask (FA>0.2). Figure 2c shows the TRT reliability values of all 4 diffusion metrics of 48 WM tracts in the JHU ICBM-DTI-81 atlas. High ICC values were observed for the 4 metrics across 48 WM tracts: FA (ICC=0.89±0.06), MD (ICC=0.80± 0.11), AD (ICC=0.80±0.11) and RD (ICC= 0.86±0.08).

**Figure 3:**
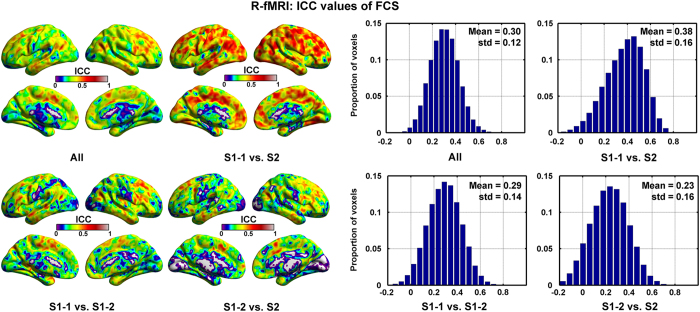
ICC values of FCS across the 3 different R-fMRI sessions. The brain figures were visualized using the BrainNet Viewer (http://www.nitrc.org/projects/bnv/)^63^.

Figure 3 exhibits the ICC maps and distribution of FCS across the whole brain between different R-fMRI sessions. The ICC maps of FCS illustrate that S1-1 and S2 have a higher TRT reliability (ICC=0.38±0.16) the others.

Table 2 illustrates the ICC values of head motion parameters across three R-fMRI scans. Most of the head motion measurements show a moderate TRT reliability between different sessions.

## Usage Notes

All data are freely available from the INDI/CoRR consortium, which can be downloaded and used for scientific research purposes. The users of this dataset should acknowledge the contributions of the original authors, properly cite this article and use these data only for scientific research purposes.

The results of image quality control are available from our group website (http://www.yonghelab.org/downloads/data), enabling users to choose data according to specific search criteria.

Notably, the imaging parameters of our diffusion MRI data are not cutting-edge compared to several other diffusion MRI sequences (e.g., diffusion spectrum imaging and high angular resolution diffusion imaging). However, for each session, our D-MRI data were repeated twice (NEX=2) to ensure a high SNR of the diffusion weighted images.

The data are shared in documented standard formats, such as NIFTI and CSV files. Therefore, all data can be further processed with many open access MRI data analysis software packages such as FSL^51^, AFNI^47^, SPM^49^, FreeSurfer^61^, NIPY^62^, GRETNA^40^, BrainNet Viewer^63^, DPARSF^54^, and PANDA^64^.

## Additional Information

**How to cite this article:** Lin, Q. *et al.* A connectivity-based test-retest dataset of multi-modal magnetic resonance imaging in young healthy adults. *Sci. Data* 2:150056 doi: 10.1038/sdata.2015.56 (2015).

## Supplementary Material



## Figures and Tables

**Table 1:** Instructions before and after MRI scan.

(1)	Do not undertake strenuous exercise on the day before the MRI scan;
(2)	Do not consume hard drinks on the day before the MRI scan;
(3)	Do not consume stimulating drinks (e.g., tea, coffee or energy drinks such as Red Bull) for 6 h before the MRI scan;
(4)	Have a good rest on the day before the MRI scan to ensure good conditions for scanning;
(5)	Lie still to rest and relax, keep motionless as possible during MRI scan;
(6)	Keep eyes closed but do not fall asleep (for R-fMRI scan);
(7)	Ask the subjects whether they kept their eyes closed or fell asleep during the R-fMRI scan (after each R-fMRI scan).

**Table 2:** ICC values of head motion parameters across different scan sessions.

	**All**	**S1-1 versus S1-2**	**S1-1 versus S2**	**S1-2 versus S2**
Mean FD	0.43	0.32	0.57	0.41
Standard DVARS	0.57	0.62	0.62	0.48
Translation	0.18	0.20	0.32	−0.035
Rotation	0.34	0.30	0.52	0.15
